# Journal club: Grey areas in paramedic decision-making for out-of-hospital cardiac arrest

**DOI:** 10.1016/j.resplu.2026.101225

**Published:** 2026-01-12

**Authors:** David Purkarthofer, Roos Edgar, Renata Roberta Dantas Silva, Erik Boberg, Sabine Nabecker

**Affiliations:** a Medical University of Graz, Graz, Austria; b Department of Health Studies, FH JOANNEUM University of Applied Sciences, Graz, Austria; c Department of Anesthesia and Intensive Care, IRCCS San Raffaele Scientific Institute, Via Olgettina 60, Milan, Italy; d Department of Cardiology, Radboud University Medical Center, Nijmegen, the Netherlands; e Graduate Program in Nursing at the Paulista School of Nursing, Federal University of São Paulo, São Paulo, Brazil; f Department of Clinical Science and Education, Södersjukhuset, Center for Resuscitation Science, Karolinska Institute, Stockholm, Sweden; g Department of Anesthesiology and Pain Management, Sinai Health System, University of Toronto, Toronto, Canada

**Figure:**
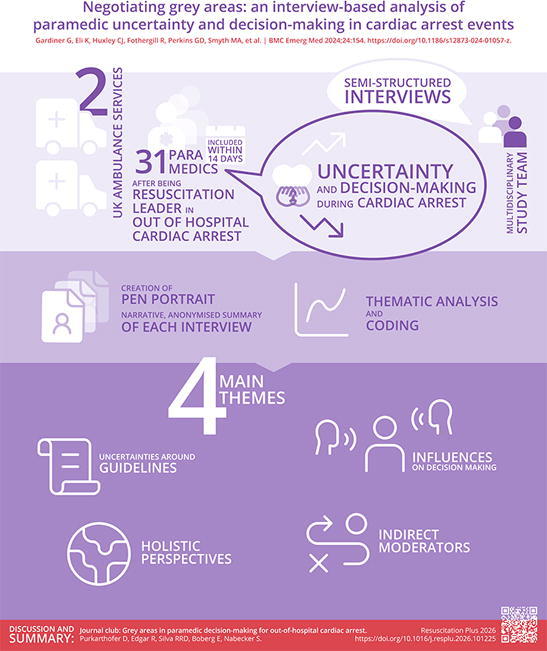

This journal club article was written as part of the 2024/25 2nd Young ERC Resuscitation Science Masterclass.^1^ It discusses the paper “Negotiating grey areas: an interview-based analysis of paramedic uncertainty and decision-making in cardiac arrest events.”^2^

## Which knowledge gap does this paper address?

Emergency medical services (EMS) provide advanced cardiopulmonary resuscitation in out-of-hospital cardiac arrests (OHCA). Successful cardiopulmonary resuscitation (CPR) requires a collaborative effort between the bystander, who witnesses a cardiac arrest and provides first resuscitation measures, the EMS, and the hospital.^3^ EMS frequently lacks crucial information like the victim’s underlying health conditions or an existing “do not attempt CPR” order. In OHCA, decision-making is especially complex, because current resuscitation and ethical guidelines,^4^, ^5^ while increasingly addressing this topic, may fail to provide effective solutions in difficult situations with limited information. The qualitative study discussed here explores in-depth factors that cause uncertainty and influence decision-making regarding treatment, transport, or termination of resuscitation (TOR) in OHCA.^2^

## What was the study’s design?

The study employed a qualitative research design using semi-structured interviews and thematic analysis,^6^, ^7^ grounded in a critical realist perspective.^8^ Recruitment targeted paramedics from two distinct Ambulance Services in the UK, from rural and urban environments, to capture diverse experiences in OHCA management. The pen portraits technique was utilised, summarising interview transcripts into narrative formats that preserved essential details for comparison and thematic identification.^9^ This approach allowed contextual understanding of participants’ decision-making processes and reflections on OHCA events. During thematic analysis, codes and themes were developed; direct quotes were used to enhance clarity. Scientific rigour was supported through reflexive note-taking, iterative review of pen portraits and themes involving a broader interdisciplinary team, transcript cross-checking, regular coding discussions, and independent double-coding of 25% of pen portraits.

## What were the key findings of the study?

In total, 31 interviews were conducted, describing 32 OHCA events. Four themes emerged from the thematic analysis of interview data. The first theme explored uncertainties in resuscitation guidelines, specifically those related to initiation and continuing resuscitation efforts. The second theme investigated personal, practical, and relational factors that influenced decision-making, which refer to the broad range of non-clinical influences that shape paramedics' decisions. These include the individual perspectives and experiences paramedics bring to a case, the situational and operational pressures they face in the moment, and the interpersonal dynamics within the team and with others on scene. The third theme described how the holistic assessment of a combination of factors influences and aids decision-making. The last theme captures factors influencing OHCA management over longer time periods or on a broader scale than the individual event, how previous experiences, further training, and feedback influence next resuscitations.

## Are there any important methodological considerations to learn from the study?

A key methodological theme in this study is the choice of interview format and mode. Qualitative interviewing is well suited to research questions that seek to understand how clinicians interpret events, weigh competing priorities, and justify decisions in context. The authors used individual telephone interviews, a defensible and thoughtful approach given the sensitivity of OHCA decision-making and the potential emotional and ethical weight associated with initiation and termination of resuscitation. Individual interviews can enhance confidentiality and psychological safety, minimise peer or hierarchical influences, and facilitate more detailed personal narratives. Conducting interviews by telephone also enhances feasibility in shift-based professions and may have enabled timely recruitment. However, telephone interviews inevitably limit access to non-verbal cues and may alter rapport and conversational depth.

An alternative design would have been focus groups, which can be particularly valuable for exploring shared norms, team culture, and how decisions are negotiated collectively on scene. Such group interaction can generate insights that individual interviews cannot, though it also introduces risks such as dominant voices or conformity shaping the discussion. Overall, the chosen approach is well aligned with the authors’ aim to elicit individual perspectives, while a focus group design might have yielded complementary insights into the social processes underpinning team-based decision-making.

## What are the most important strengths and limitations of the study?

The authors followed an established qualitative method to describe paramedic uncertainty and decision-making in OHCA. This is a complex topic, and the results are highly dependent on the context. A strength of the study is that the authors recruited a good number of paramedics, both from rural and urban areas, to support the themes they found in the thematic analysis. Furthermore, OHCA events were described in sufficient detail, and the authors reported that saturation was reached.^10^ The authors could have provided more demographic information about the interviewed paramedics, as it is now difficult to assess their personal background (e.g., gender, ethnicity, age). For triangulation purposes, some baseline characteristics would have been helpful to interpret the results. In addition, the authors could have explored and compared differences between urban and rural areas and reported any divergent findings. Another strength was the patient and public involvement panel that was used to inform the study development and evaluation process. Generalizability of the results to other cultural backgrounds, lower-resource settings, or even to other EMS systems outside of the UK may be limited. It would have been useful if the authors had published the specific interview guide with the manuscript, to enable replication of the study in different areas of the world and in different contexts.

Finally, future studies would benefit from a more detailed description of the interviewers, as the background of the research team can meaningfully shape qualitative data collection and interpretation.^11^ Providing information about the interviewers’ and coders’ experience with OHCA, for example, would help readers better understand the lens through which the data were gathered and analysed.

## How will the result affect your clinical practice?

This study reinforces that ambiguity is inherent to OHCA care, particularly when deciding whether to initiate or terminate resuscitation. Its emphasis on team-based and holistic decision-making aligns closely with the updated ERC guidelines, which now highlight that TOR decisions should be made collectively and with consideration of the wider clinical and contextual picture.^5^ Explicitly acknowledging uncertainty, as the study suggests, may help teams build tolerance for residual ambiguity while still working within guideline-based frameworks.

For practice and training, the findings from Gardiner and colleagues support the need for scenario-based education that incorporates not only clinical criteria but also ethical tensions, emotional stressors, and team communication. The study’s qualitative insights into decision-making complement the ERC guidelines’ predominantly medical and quantitative focus and may help clinicians and educators better understand how TOR decisions unfold in real-world settings.

## What do you see as the next steps in research?

Further studies exploring uncertainty and decision-making in OHCA are needed, with different cultural backgrounds, from low-income settings, different professions, and different EMS systems. If this is achieved, combining the data from multiple studies could increase generalizability, for example, if a qualitative synthesis is performed. Performing similar studies but focusing on the whole team as decision-makers, with group interviews, could be beneficial to get an even more nuanced picture of factors influencing the decision-making process in OHCA. Finally, as this study shows that uncertainties are omnipresent, future studies should explore possible interventions to mitigate these uncertainties, followed by patient outcome studies to investigate if such interventions have the desired effect.

## Ethical considerations

Not applicable.

## CRediT authorship contribution statement

**David Purkarthofer:** Writing – review & editing, Writing – original draft, Visualization, Conceptualization. **Roos Edgar:** Writing – review & editing, Writing – original draft, Conceptualization. **Renata Roberta Dantas Silva:** Writing – review & editing, Writing – original draft, Conceptualization. **Erik Boberg:** Writing – review & editing, Writing – original draft, Conceptualization. **Sabine Nabecker:** Writing – review & editing, Supervision, Conceptualization.

## Declaration of competing interest

The authors declare the following financial interests/personal relationships which may be considered as potential competing interests: “Roos Edgar: Young investigator editorial board member Resuscitation Plus. The other authors declare no competing interests.”.
